# An unusual presentation of Moyamoya disease managed conservatively in a resource limited region: A case report

**DOI:** 10.1016/j.radcr.2026.07.101

**Published:** 2026-09-01

**Authors:** Zareen Tabassum, Md. Deluwar Hussen, Afsana Akter Mimi, Arefa Insan Momo, Sharmin Akter Riya, Munim Hasan Choudhury, Sayema Begum Jahra, Anika Tasnim Khan

**Affiliations:** a Medical officer, Department of internal medicine, Dinajpur medical college, Dinajpur 5200, Bangladesh; b Mymensingh Medical College, Mymensingh, Bangladesh; c Rangpur Medical College, Rangpur, Bangladesh; d Chittagong Medical College, Cumilla, Bangladesh; e Anwer Khan Modern Medical College Hospital, Dhaka, Bangladesh; f Sylhet Womens Medical College and Hospital, Sylhet, Bangladesh; g Medical Officer, Obs and Gynae, Chittagong Medical College, Chattogram 4203, Bangladesh

**Keywords:** Pediatric, Moyamoya disease, Blindness, Under-developed countries, Case report

## Abstract

Moyamoya disease is a rare cerebrovascular disease of chronic and progressive nature, characterized by stenosis and occlusion of the Circle of Willis vessels and formation of compensatory collateral circulation. Here we have reported a case of a 10 year old male patient who first developed seizure, hemiparesis 1 year ago, followed by deteriorating hemiparesis, prolonged fever and blindness. Although seizure hemiparesis are common presentations of the disease, blindness and prolonged fever have been rarely reported. The diagnosis in this case was established by Magnetic Resonance Imaging (MRI) and Magnetic Resonance Angiogram (MRA), where vessel occlusion, encephalomalacia and compensatory circulation formation were detected. Due to financial hardships and limitations of the surgical settings in the hospital, conservative treatment was provided. In fact, there has been no report of performing revascularization surgery in a patient with Moyamoya disease in Bangladesh. This case highlights that progressive cortical blindness and posterior circulation involvement may occur in pediatric Moyamoya disease. In resource-limited settings, characteristic MRI and MRA findings can facilitate early diagnosis when Digital Subtraction Angiography is unavailable, although definitive management with revascularization surgery remains the preferred treatment.

## Introduction

Moyamoya disease is a rare, chronic cerebrovascular disease that is characterized by progressive steno-occlusive changes in the terminal portion of the Internal Carotid Artery and/or its proximal branches, leading to a compensatory network of collateral vessels at the base of the brain, depicting the hazy puff of smoke seen on imaging [1]. The disease has a bimodal distribution of incidence, peaking at age 5 and age 40 [2]. Prevalence is higher in East Asia, affecting mostly women [3]. Patients usually present with Transient Ischaemic Attack/Stroke-like features, headache, seizure and are mainly diagnosed by Digital Subtraction Angiogram (DSA), Magnetic Resonance Imaging (PWI or MRA), CT angiogram, Single Photon Emission Computerized Tomography (SPECT) [4]. Treatment options include pharmacotherapy and surgical intervention. Revascularization surgery is the first line treatment [5]. Here we are reporting a case of a 10-year-old boy with Moyamoya disease having uncommon presentations with blindness and prolonged fever. The study focuses on helping doctors working in resource-limited settings to be more vigilant about detecting unusual or confusing symptoms and to investigate if those are pointing towards an underlying rare disease. This may help in early diagnosis and/or better management of the case.

## Case presentation

**Fig. 1:**
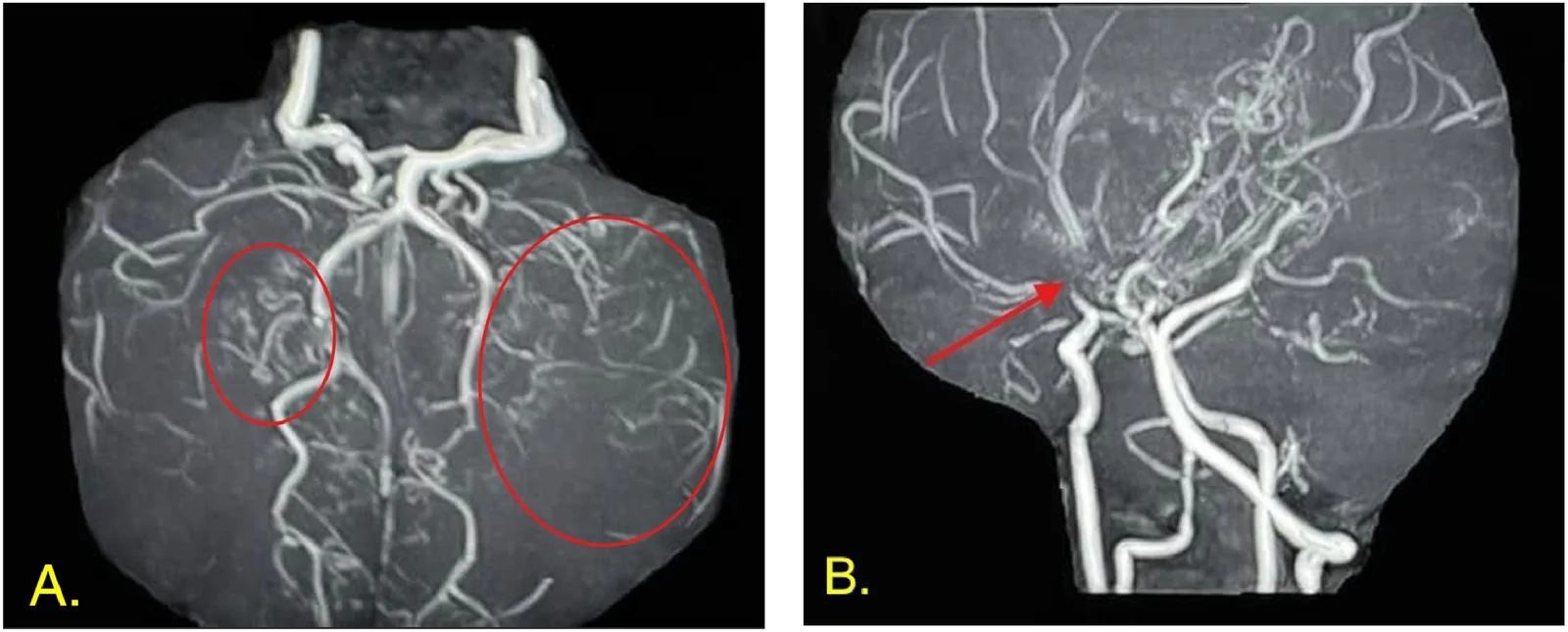
(A) Coronal MRA demonstrating bilateral marked stenosis of the terminal ICAs with extensive collateral vessel formation producing the characteristic “puff of smoke” appearance (red circles). (B) Sagittal MRA showing marked steno-occlusive changes localized to the terminal portions of the ICA (red arrow).

**Fig. 2:**
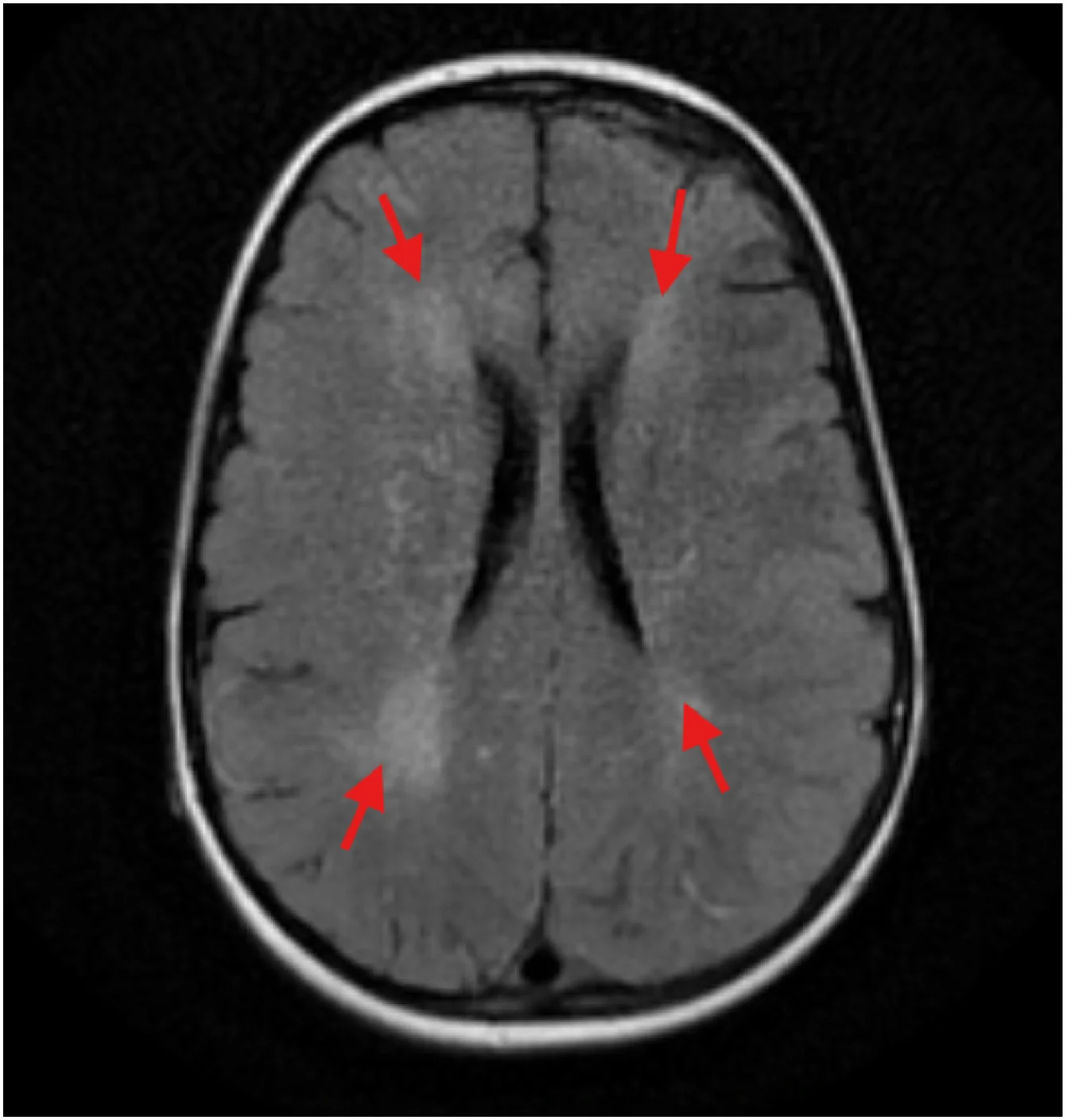
Axial fluid-attenuated inversion recovery (FLAIR) magnetic resonance imaging of the brain. Hyperintense signal abnormalities with associated encephalomalacia and gliotic changes are observed in the bilateral parieto-occipital and frontal lobes, consistent with chronic ischemic infarctions secondary to Moyamoya vasculopathy.

**Fig. 3:**
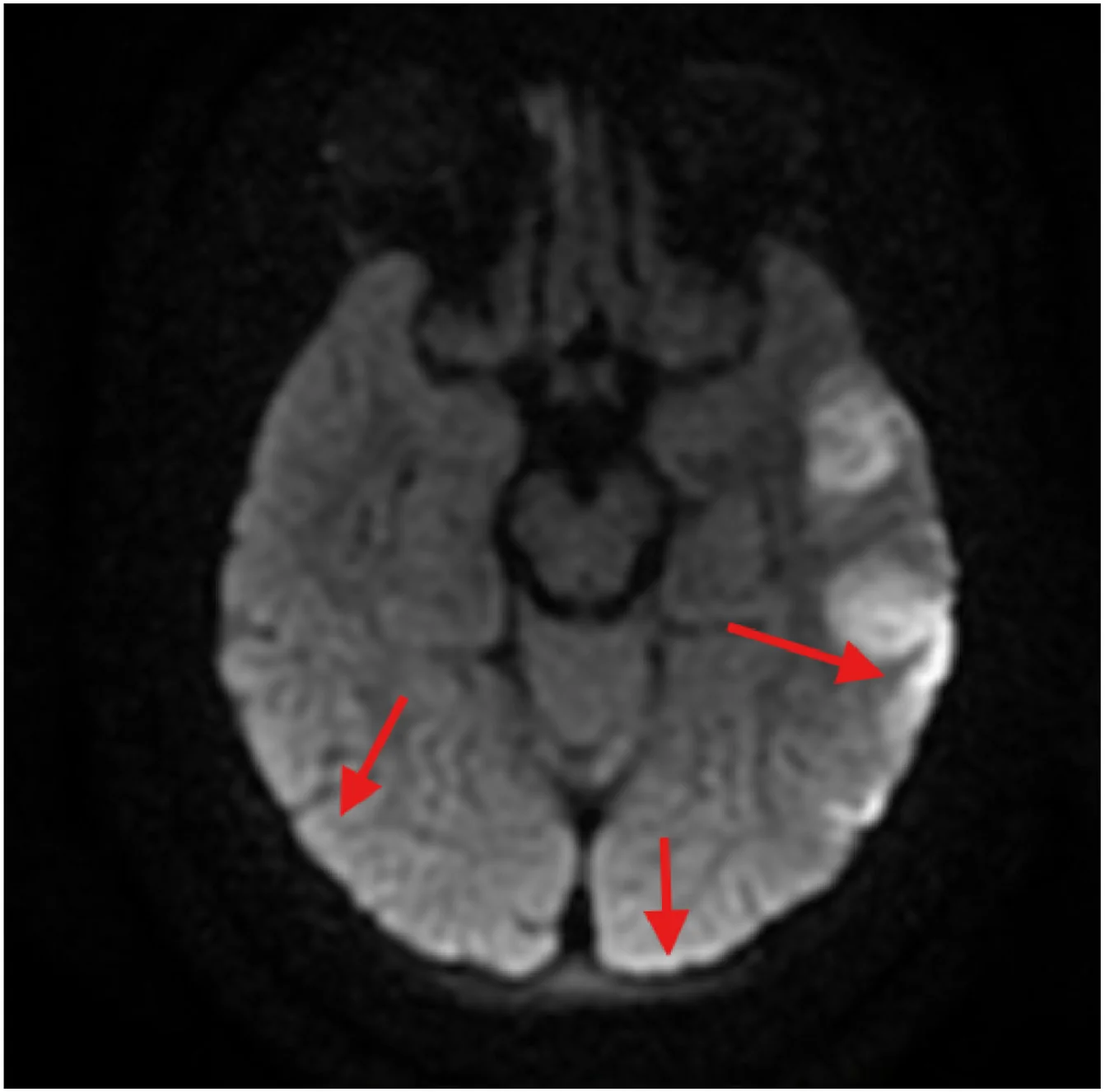
Axial diffusion-weighted imaging (DWI) of the brain. The images demonstrate areas of high signal intensity involving the bilateral parieto-occipital cortical regions with corresponding ADC reduction, consistent with acute ischemic infarction secondary to Moyamoya vasculopathy.

A 10 year old, 25 kg, male patient with no notable prior medical history, presented to the Medicine outpatient department with complaints of prolonged fever, left sided weakness of the body for the past year, loss of vision in both eyes for 5 days and 2 episodes of seizure a day before. The weakness of the body gradually advanced over the year, while visual disturbance started a month ago and ultimately led to complete blindness. The patient had a history of seizures 1 year ago for which he was prescribed Sodium Valproate at a rural Primary Healthcare Complex. Other than that, the incidence of head injury or association of headache, loss of consciousness or vomiting with fever was not reported. There was no family history of stroke or ischemic heart disease. On examination, the patient appeared exhausted with normal vitals, blood pressure, cardiovascular findings and no sign of meningeal irritation. Examination of the eyes revealed a bilateral absent pupillary reflex. Neurological examination demonstrated weak movement of left sided limbs, muscle power was 4/5 according to the Medical Research Council (MRC) muscle strength scale, with increased muscle tone, 3+ patellar reflex and positive Babinski sign on the left side. The pattern of visual loss, together with bilateral parieto-occipital infarction demonstrated on MRI, was most consistent with cortical blindness. Although formal ophthalmologic examination and visual evoked potential testing were not available, no clinical findings suggested primary ocular pathology. Laboratory investigations revealed normal findings on Complete Blood Count with Erythrocyte Sedimentation Rate, Lipid profile, Serum electrolytes, Random blood sugar, Prothrombin Time with International Normalized Ratio. Cerebrospinal fluid (CSF) analysis demonstrated clear, colorless fluid with normal opening pressure, normal protein and glucose levels, and no pleocytosis or evidence of infection, suggesting no features of meningoencephalitis. No abnormalities were detected on Chest X ray or the Echocardiogram either. EEG was found to have background waves of the brain at 8-9 Hz and increased sharp and slow waves that were distributed uniformly on both sides. The recordings indicated cortical irritability associated with Moyamoya disease. The old infarctions resulted in encephalomalacia of the Parietooccipital lobes and Frontal lobes as demonstrated by MRI. Axial FLAIR imaging demonstrated hyperintense signal abnormalities with associated encephalomalacia and gliotic changes in the bilateral parieto-occipital and frontal lobes, consistent with chronic ischemic infarctions secondary to Moyamoya vasculopathy (Fig. 2). Additionally, axial DWI revealed areas of high signal intensity involving the bilateral parieto-occipital cortical regions with corresponding ADC reduction, suggestive of acute ischemic infarction (Fig. 3). The Magnetic Resonance Angiography revealed that the brain had several swollen vessels, which were noted to produce a puff of smoke effect (Fig. 1). The stenotic areas were found to be the highest parts of both Internal Carotid Arteries (ICAs) as well as the Middle (MCAs) and Posterior Cerebral Arteries (PCAs). Overall, Venous Magnetic Resonance Venography (MRV) studies were normal except that the transverse sinus showed a slight asymmetry. The venous system was not thrombotic or obstructed.

The patient was managed conservatively with tablet Sodium Valproate (300 mg) daily at night and tablet Clonazepam (0.5 mg) daily at night for seizure control, along with tablet Aspirin (75 mg) once daily for secondary stroke prevention. Levetiracetam (250 mg) twice daily was added for seizure control and neurovascular support. Physiotherapy, nutritional support, and rehabilitation measures were provided to address left-sided weakness and functional limitations. Although revascularization surgery represents the preferred definitive treatment for symptomatic Moyamoya disease, the procedure could not be performed in this patient due to significant socioeconomic limitations and limited availability of specialized cerebrovascular surgical facilities. The patient and family were advised regarding referral to a higher-level center for neurosurgical evaluation and possible revascularization; however, they opted to continue conservative management because of financial constraints. The patient was discharged with advice to continue tablet Sodium Valproate (300 mg) daily at night and regular physical therapy. On a follow up after 1 month, the patient's seizure frequency reduced after modifying treatments and small motor improvement developed with the help of physical therapy. But the patient showed no change in his permanent vision impairment of both eyes.

## Discussion

Moyamoya disease mainly involves bilateral distal segments of intracranial internal carotid arteries and proximal portions of Anterior Cerebral Artery and Middle Cerebral Artery; frequent involvement of PCA has also been reported, but the posterior circulation acts as a source of collateral circulation as well, so symptoms seldom develop [6,7]. Progressive nature of the disease gradually results in the release of angiogenic factors and the development of collateral vessels, giving off the “puff of smoke” appearance seen on imaging [8]. In our case, the patient not only had ACA, MCA, and PCA involvement but also showed encephalomalacia of areas supplied by the posterior circulation. This is a rare finding in itself, as posterior areas of the brain are scarcely affected in MMD cases.

MMD is mostly prevalent in East Asia, with the majority of the cases being reported in Japan (prevalence is 10.5 per 100,000) and only a few in Bangladesh [9]. So this report aims to contribute to the enrichment of the gap in knowledge regarding MMD in Bangladesh.

MMD commonly produces symptoms like hemiparesis, seizures, facial asymmetry, speech disturbance and headache [10]. Lower body weakness, visual disturbance, loss of consciousness and involuntary movements are some of the uncommon manifestations [3]. Our case presented with an uncommon symptom that was blindness and prolonged fever. According to a study using the National Inpatient System Database, only 3.7% MMD cases develop visual disturbances. Among different races included in the study, 13.3% Asians had some form of visual abnormality [11]. In our patient, the disease first produced seizure and weakness 1 year ago. As the patient remained undiagnosed, the disease then progressed to the development of hemiparesis, blindness and further seizure attacks.

Visual impairment in Moyamoya disease may result from retinal ischemia, optic pathway involvement, or cortical blindness caused by bilateral occipital infarction. In our patient, bilateral parieto-occipital encephalomalacia strongly supported cortical blindness as the underlying mechanism. Unfortunately, comprehensive ophthalmological evaluation and electrophysiological testing could not be performed because of resource limitations

Although fever has occasionally been reported in pediatric Moyamoya disease, its pathophysiological relationship remains uncertain. Previous reports, including Khalesi et al. [12] described several children initially suspected of encephalitis because of fever and seizures [12].

For fever and seizure evaluation, we initially performed CBC with ESR, Serum electrolytes and Random Blood Glucose level. Normal findings of the mentioned investigations excluded Meningoencephalitis, Electrolyte imbalance, Hypoglycemia. To evaluate left sided hemiparesis, MRI and MRA of the brain were advised where frontal and parieto-occipital encephalomalacia, along with steno-occlusive changes of cerebral vessels and formation of collateral vessels were detected. This established the diagnosis of ischemic stroke due to Moyamoya Disease. To explore atherosclerotic and cardiovascular causes, Serum lipid profile, Chest X-ray and echocardiogram were performed, but the findings were normal. Although Digital Subtraction Angiography remains the gold standard for diagnosing Moyamoya disease and surgical planning, it could not be performed because of financial limitations and limited local availability. Diagnosis was therefore established using characteristic MRI and MRA findings. Nevertheless, inflammatory vasculopathies, autoimmune disorders, and secondary Moyamoya syndrome should also be considered in patients with posterior circulation abnormalities. Because Digital Subtraction Angiography and comprehensive autoimmune investigations were unavailable, these possibilities cannot be completely excluded.

In patients who are not surgical candidates or where revascularization cannot be immediately performed, medical management may be considered as a supportive approach to reduce the risk of ischemic events and control associated symptoms. However, conservative therapy does not halt the progressive vasculopathy, and close clinical and radiological follow-up remains essential. In our patient, revascularization was considered the optimal treatment strategy; however, socioeconomic barriers and limited access to specialized surgical facilities prevented definitive intervention. Both Aspirin and Clopidogrel are used in pediatric cases, but the efficacy and survival improvement require further evaluation to establish stronger evidence [8,13]. Cortical artery revascularization, on the other hand, is the mainstay treatment due to its role in reducing the recurrence rate of ischemic attacks and improving outcomes. Revascularization surgery is done by Direct and Indirect methods [14]. To the best of our knowledge, published reports describing revascularization surgery for pediatric Moyamoya disease in Bangladesh remain extremely limited [15]. The hospital did not have this specific surgical facility, the patient was advised to go to a higher center for further investigation and intervention. But due to financial limitations, the patient opted for continuing conservative treatment.

Despite small improvements noticed after treatment of the patient, long term outcome of a late diagnosed and advanced case getting conservatively treated is subjected to further monitoring.

Lack of awareness about rare diseases, late diagnosis and financial burden act as obstacles in the way of proper reporting and management of these diseases. In our case, although the symptoms pointed towards meningoencephalitis like presentation, clinical and laboratory tests suggested otherwise. So, we must investigate with vigilance to look for unusual diseases before starting treatment. In resource-limited and financially poorer regions, clinical correlation becomes more important than investigations.

## Conclusion

This case report aims to create awareness among physicians in low-resource settings regarding the importance of early diagnosis of a rare cerebrovascular pediatric Moyamoya disease and initial line of treatment when surgery is not available to prevent ischemic stroke and further neurological damage. While noninvasive MRI and MRA provide clues by demonstrating steno-occlusive vessels and collateral formation, Digital Subtraction Angiography is still the gold standard for definitive diagnosis and planning of revascularization surgery. Due to the financial and accessibility challenges in the setting of Bangladesh, the patient had to be managed conservatively with close monitoring and follow-up.

## Author contributions

ZT drafted the original manuscript, including the abstract, introduction, case presentation and discussion. MDH formulated the study concept and design, data acquisition and clinical evaluation. AAM authored the conclusion, selection of keywords, performed significant language refinement and scientific editing, prepared and annotated the figures. AIM conducted literature searches via PubMed, reviewed the manuscript, gave scientific input and performed language refinement. MDH also contributed to the literature review and critical revision of the manuscript. All authors have read and approved the final version of the manuscript.

## Patient consent

Informed consent was collected from the patient before collecting data and participation was completely voluntary.

## Appendix Supplementary materials

Image, application 1
