# Supplementary material for: An unusual presentation of Moyamoya disease managed conservatively in a resource limited region: A case report
Source: Radiol Case Rep. 2026 Sep 1;21(11):5766–70. doi: 10.1016/j.radcr.2026.07.101 (PMC13571123; doi:10.1016/j.radcr.2026.07.101)
Supplement: Supplementary file 1 [file mmc1.docx]

**Supplementary information**

**Author’s information:**

1.Dr. Zareen Tabassum, MBBS, Dinajpur Medical College, Dinajpur, Full Address: 4/1/B Golden street, Ring Road, Shyamoli, Dhaka-1207, [ztaunima72@gmail.com](mailto:ztaunima72@gmail.com), +8801953899591

2.Dr. Md. Deluwar Hussen, MBBS, Dinajpur Medical College, Dinajpur, Full Address: Ruksana Monji, Shantibagh R/A, Sreemangal-3210, Moulvibazar, Sylhet, deluwar664@gmail.com ORCID: 0009-0002-1518-5805, +8801721248551

3) Dr. Afsana Akter Mimi, MBBS, Mymensingh Medical College, Mymensingh, Full Address: Ekota Nibash, 75/13, Balughat Bazar, Dhaka Cantonment-1206, Dhaka, [mmcafsanamimi@gmail.com](mailto:mmcafsanamimi@gmail.com), +8801814170146

4) Dr. Arefa Insan Momo, MBBS, Rangpur Medical College, Rangpur, Full Address: 48/7 R.K. Mission Road,Gobibagh, Dhaka-1203, [arefainsanmomo@gmail.com](mailto:arefainsanmomo@gmail.com), +8801706913795

5) Dr. Sharmin Akter Riya, MBBS, Chittagong Medical College, Chittagong, Full Address: Rampur, Kutwali Model, Shimra-3500, Cumilla, [sharminakteriom35@gmail.com](mailto:sharminakteriom@gmail.com), +8801521718321

6) Dr. Munim Hasan Choudhury, MBBS, Anwer Khan Modern Medical College Hospital, Dhaka, Bangladesh, Full address: House No- 9/B-2, Hare Street, Wari, Dhaka-1203, drmunim.46@gmail.com ORCID ID: 0009-0006-0097-857X, +8801689545641

7) Dr.Sayema Begum Jahra, MBBS, Sylhet Womens Medical College and Hospital, Sylhet, Full address:19, Al-amin Road, Pathantula, Sylhet-3100, sayemabegum321@gmail.com, ORCID ID: 0009-0009-6364-3349 ,+880 1751166244

8) Dr. Anika Tasnim Khan, MBBS, Chattogram Medical College ,Chattogram, Bangladesh, Address: Chattogram, Bangladesh [anikatasnimkhan195@gmail.com](mailto:anikatasnimkhan195@gmail.com), Phone no: +1(945)402-7017,ORCHID ID: 0009-0005-5499-9598

**Corresponding author:**

Dr. Md. Deluwar Hussen, MBBS, Dinajpur Medical college, Dinajpur, Full address: Ruksana Monji, Shantibagh R/A, Sreemangal-3210, Moulvibazar, Sylhet, deluwar664@gmail.com ORCID ID: 0009-0002-1518-5805, +8801721248551

**Declaration of interest:**

All authors confirm they adopt no conflict of interest and would like to support the corresponding author for submission.

**Informed Consent:**

Informed consent was collected from the patient before collecting data and participation was completely voluntary.

**Funding information:**

This research received no funding from any agent in the public, commercial or the hospital authority.

**Data sharing statement:**

No separate datasets were generated or analyzed for this case report beyond the clinical details presented in the manuscript. Any additional information, if requested for legitimate academic or clinical purposes, may be made available by the corresponding author upon reasonable request, subject to institutional approval and strict adherence to patient confidentiality and data protection standards. Identifiable information will not be shared, and any disclosure will be limited to fully anonymized content in accordance with ethical guidelines and informed consent provisions.

**Ethical Considerations:**

Institutional Review Board approval was not required for this case report in accordance with institutional policies. Written informed consent was obtained from the patient for publication of clinical details and images. The authors declare no conflicts of interest, and all ethical standards were adhered to throughout the preparation of this manuscript.

**Institutional Affiliation:**

Dream USMLE was the primary institution for this research, ensuring ethical guidance, protection of data and compliance with regulations.

**Acknowledgments:**

We are especially grateful to Dr. Abdullah Al Masud, MD, FACP and his organization Dream USMLE, 16523 Wildhorse Creek 63017, Chesterfield, MO, USA, who gave us the technical guidance.

**Registration:**

It is not applicable for this study.

**Reporting Guideline:**

This manuscript was prepared in accordance with the CARE (CAse REport) guidelines to ensure transparent, complete, and standardized reporting of this case.

**Generative AI use**: For paraphrasing of citations and basic structure of outline, actually very limited use of Quillbot has been made and all have been edited properly after review.

**Author Contributions:**

ZT drafted the original manuscript, including the abstract, introduction, case presentation and discussion. MDH formulated the study concept and design, data acquisition and clinical evaluation. AAM authored the conclusion, selection of keywords, performed significant language refinement and scientific editing, prepared and annotated the figures. AIM conducted literature searches via PubMed, reviewed the manuscript, gave scientific input and performed language refinement. MDH also contributed to the literature review and critical revision of the manuscript. All authors have read and approved the final version of the manuscript.
